# Efficacy and safety of the single-dose pegylated G-CSF vs. daily G-CSF for peripheral blood stem cells mobilization in donors: a multicenter, randomized controlled trial

**DOI:** 10.3389/fimmu.2026.1737252

**Published:** 2026-06-24

**Authors:** Jiali Li, Sha Zhou, Xiaoping Li, Xiangyu Ma, Sanbin Wang, Yicheng Zhang, Shifeng Lou, Jun Rao, Ping Wang, Lidan Zhu, Ting Chen, Xixi Xiang, Shichun Gao, Han Yao, Peiyan Kong, Lei Gao, Cheng Zhang, Xi Zhang, Li Gao

**Affiliations:** 1 Medical Center of Hematology, Institute of Science Innovation for Blood Ecology and Intelligent Cells, The Second Affiliated Hospital of Army Medical University, Chongqing, China; 2 Chongqing Key Laboratory of Hematology and Microenvironment, Chongqing, China; 3 State Key Laboratory of Trauma and Chemical Poisoning, Army Medical University, Chongqing, China; 4 Department of Hematology, 920th Hospital of Joint Logistics Support Force, Kunming, China; 5 Department of Epidemiology, College of Preventive Medicine, Army Military Medical University, Chongqing, China; 6 Department of Hematology, Tongji Hospital, Tongji Medical College, Huazhong University of Science and Technology, Wuhan, China; 7 Department of Hematology, The Second Affiliated Hospital of Chongqing Medical University, Chongqing, China

**Keywords:** allo-HSCT, efficacy, mobilization, Peg-G-CSF, randomized clinical trial

## Abstract

**Background:**

The mobilization of peripheral blood stem cells(PBSC) needs daily injection of granulocyte colony stimulating factor (G-CSF), which brings inconvenience to healthy donors. Can pegylated granulocyte colony-stimulating factor (Peg-G-CSF) solve this problem by single dose injection?

**Methods:**

This multicenter, randomized controlled study was conducted from May 2018 to April 2019. Eligible donors were randomly selected to received treatment with 12 mg Peg-G-CSF on day 1 or 10 µg/kg/d G-CSF consecutively from day 1. PBSC apheresis of the donors was conducted on day 5. The primary endpoint was the percentage of donors who collected ≥4×10^6^ CD34^+^cells/kg recipient weight after single apheresis.

**Results:**

Eighty (83.3%) of 96 donors in the Peg-G-SCF group and 76 (79.2%) of 96 donors in the G-CSF group collected ≥4×10^6^ CD34^+^cells/kg recipient weight. The peak value of circulating CD34^+^ cell count occurred on day 5 in both groups. The median yield of CD34^+^ cell collected in the Peg-G-CSF group was comparable to that in the G-CSF group by a single apheresis procedure. Moreover, the incidence of side effects in both groups was similarly, no significant difference was observed in engraftment, graft-versus-host disease (GVHD) or survival between the two groups of recipients. By multivariate analysis, The CD34^+^ cell count on the fifth day was the variable that may have significantly affected the CD34^+^ yield.

**Conclusion:**

Mobilization via a single injection of Peg-G-CSF can match traditional G-CSF mobilization in terms of efficacy and safety and is expected to provide a better alternative to traditional G-CSF mobilization.

**Clinical Trial Registration:**

www.chictr.org.cn, identifier ChiCTR1800015716

## 1 Introduction

Granulocyte colony-stimulating factor (G-CSF) is a conventional agent for inducing egress of stem/progenitor cells from their niche(s), primarily in bone marrow, to the peripheral blood (1, 2). For mobilization, due to a short half-life, G-CSF requires daily injections (3), which is inconvenient and greatly increases donor discomfort.

Pegylated granulocyte colony-stimulating factor (Peg-G-CSF), is a polyethylene glycol-conjugated form of G-CSF (4). The special pharmacokinetic properties of Peg-G-CSF result in decreased serum clearance as well as reduced renal excretion and ultimately lead to a relatively long half-life (from 15 to 80 h) after subcutaneous injection (5). As a long-acting peripheral blood stem cell (PBSC) mobilization agent, compared with traditional drugs, Peg-G-CSF has been confirmed to lead to shorter hospitalization durations and fewer side effects in many autogenic transplantation trials (6–8). A single dose of Peg-G-CSF has also preliminarily shown potency equal to that of G-CSF with regard to CD34^+^ cell mobilization and yield in cases of allogeneic hematopoietic stem cell transplantation (allo-HSCT) (9). Moreover, it was reported that Peg-G-CSF may reduce graft-versus-host disease (GVHD) while retaining graft versus leukemia (GVL), which offers an attractive advantage in animal study (10). Previous studies on allo-HSCT have all been single-arm studies with small sample sizes. We have reported the efficiency and safety in 146 donors from four HSCT centers in China in a retrospective study (11). However, a randomized control trial is needed to rigorously confirm the efficacy and safety of Peg-G-CSF versus traditional G-CSF for allo-HSCT mobilization.

Therefore, for the first time, we designed an open-label, multicenter, randomized controlled trial to comprehensively compare the efficacy and safety of Peg-G-CSF and G-CSF for mobilization in healthy donors as well as to compare the outcome of allo-HSCT in recipients.

## 2 Methods

### 2.1 Study design and donor enrollment

The sample size estimation was calculated based on the primary outcome of healthy donor mobilization efficacy after a single leukapheresis procedure. Optimal mobilization was performed in accordance with the recommendation of the American Society for Blood and Marrow Transplantation: CD34^+^cell counts ≥4×10^6^/kg recipient weight by a single apheresis procedure (12). In accordance with our single center data in 2017, the mobilization efficacies of CD34^+^cell counts ≥4×10^6^/kg recipient weight after a single leukapheresis procedure of Peg-G-CSF and G-CSF were 0.9 and 0.7, respectively. A sample size of a total of 184 recipients was obtained with a two-sided type I error rate of 0.05 and a statistical power of 0.8 (no donors were included in the preliminary experiments in this study). Considering the possible loss of donors to follow-up (5%), the final enrolment target was 194 donors (97 in each treatment group). All the healthy donors were randomly divided into the Peg-G-CSF and the G-CSF groups at a 1:1 ratio with STATA package (StataCorp, College Station, TX).

**Figure 1:**
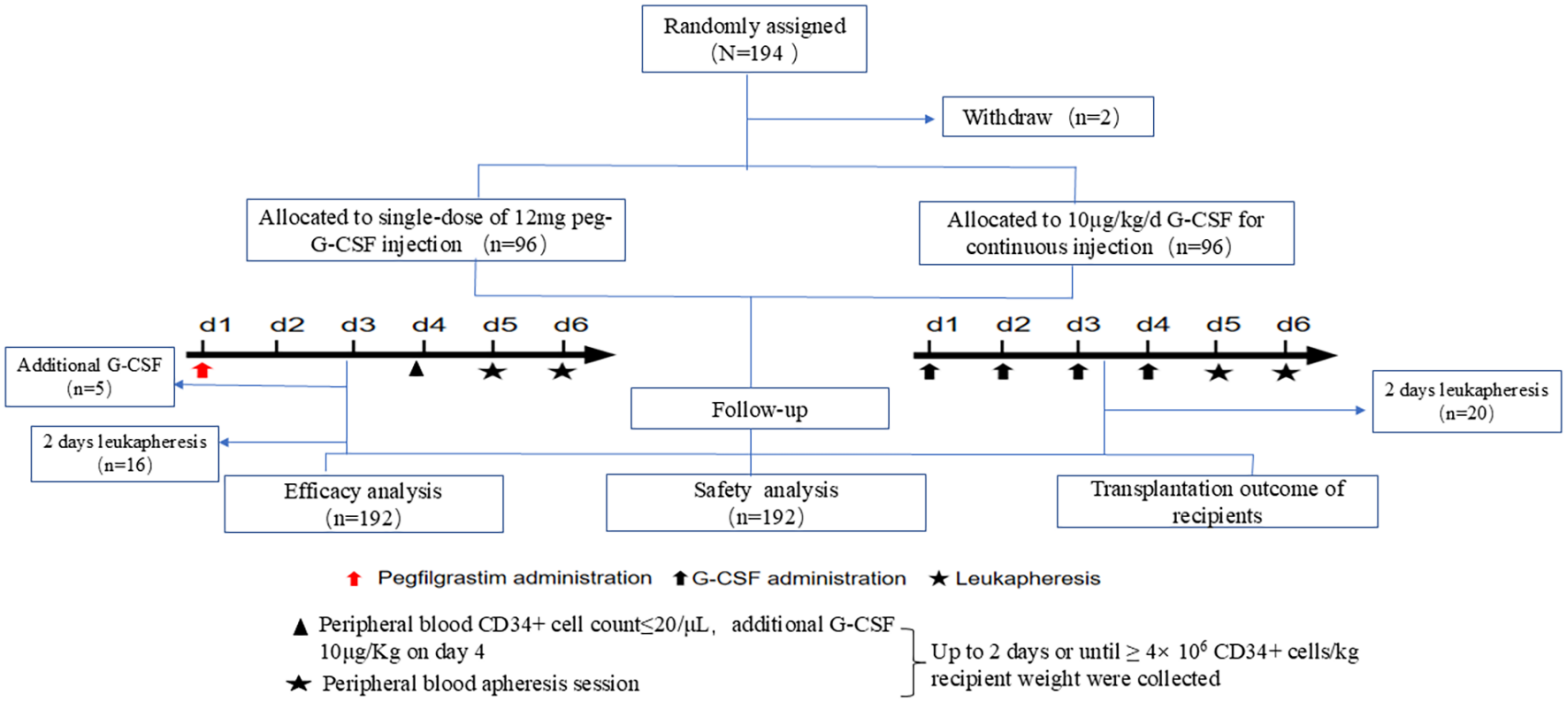
Procedure flow chart. Single-dose of 12 mg Peg-G-CSF and consecutive 10 ug/kg/d G-CSF were given to donors of the two groups. Leukapheresis was performed on day 5. If the peripheral CD34^+^cell count was <4×10^6^/Kg recipient weight, additional leukapheresis was administered on day 6.

Healthy donor relatives with their corresponding acute leukemia recipients who needed to undergo allo-HSCT were enrolled from 4 transplant centers in China (**Figure 1**). The primary endpoint of this trial was the percentage of donors collected CD34^+^cells ≥4×10 (6)/kg recipient weight after a single apheresis procedure. The secondary endpoints included white blood cell (WBC) and monocyte count, the yields of mononuclear cell and CD34^+^cell after collection, adverse events (AEs) in donors after drug administration, the lymphocyte subtype in graft. Engraftment, GVHD and relapse incidence, overall survival (OS), and progression-free survival (PFS) of recipients after allo-HSCT were also monitored.

Eligible donors met the following criteria: voluntary participants who were related to the allo-HSCT recipients; age of 18–55 years; weight of 50 ~ 100 kg; normal heart, liver and kidney functions; negative results from the tests for hepatitis B surface antigen and core antibody, or negative HBV-DNA patients with negative hepatitis B surface antigen and positive core antibody; negative cytomegalovirus PCR test results, and normal ECG and abdomen and chest ultrasound findings. All recipients were diagnosed with acute leukemia. The written informed consent forms were signed by all donors and recipients in accordance with the Declaration of Helsinki. Ethics approved through China Ethics Committee of Registering Clinical Trials (ChiECRCT-20180036). Clinical Trial number: ChiCTR1800015716 (https://www.chictr.org.cn/showproj.html?proj=25876). The study adhered to consort guidelines and the completed wand flow chart was summarized as **Figure 1**.

### 2.2 Mobilization and collection of peripheral blood stem cells

Donors in the Peg-G-CSF group were administered a single subcutaneous injection of 12 mg Peg-G-CSF (Qilu Pharmaceutical Co., Ltd, Shandong, China) on day 1, whereas donors in the G-CSF group were given subcutaneous injection of 10 µg/kg/d G-CSF (Qilu Pharmaceutical Co., Ltd, Shandong, China) every day from the first day. If the peripheral CD34^+^ cell count was ≤20/µL in the Peg-G-CSF group on day 4, the donors were temporarily given an additional 10 µg/kg G-CSF (13). Leukapheresis was performed on day 5 for both groups (**Figure 1**). Blood parameters and peripheral CD34^+^ cell count were measured on day 4 and day 5 after mobilization.

The COBE Spectra and COM.TEC systems were used for the collection of peripheral blood progenitor cells. If the collection target was reached ≥4×10 (6) CD34^+^ cells/kg recipient weight, apheresis was performed only on day 5. Otherwise, additional hematopoietic stem cells (HSCs) were harvested on day 6. HSCs collection was performed using standard institutional apheresis procedures (3 blood volumes ±10%). Generally, approximately 100 ~ 200 mL of PBSCs were collected after a single leukapheresis procedure.

### 2.3 Transplantation procedure

Recipients undergoing HLA-matched HSCT were conditioned with busulfan (Bu) and cyclophosphamide (Cy). Recipients undergoing haploidentical HSCT (haplo-HSCT) were conditioned with semustine, arabinosylcytosine (Ara-c), Bu, CY and antithymocyte globulin (ATG, Sanofi, SangStat, Lyon, France). The prophylaxis for GVHD included mycophenolate mofetil, tacrolimus, cyclosporin A, and short-term methotrexate. Supportive care was given according to standard institutional operating procedures.

### 2.4 Flow cytometry for CD34^+^ cell counting and lymphocyte subsets

We used dual platform for our CD34 counting, and we followed ISHAGE gating for CD34 detection. CD34^+^ cells in peripheral blood and grafts were stained with PE anti-human CD34^+^ and APC-Cy7 anti-human CD45 antibodies. Lymphocyte subsets were contained with surface markers and then fixed and permeabilized as previously described (14). The antibodies used for lymphocyte subset analysis were as follows: CD3+ T cells, CD3-CD19+ B cells, CD3+CD4+ T helper cells, CD3+CD8+ cytotoxic T cells, CD3+CD4+CD127low/-CD25+regulatoryTcells (Tregs), CD3-CD16+/CD56+ natural killer (NK) cells and CD45+CD19+CD24hi+CD27+CD1d+ regulatory B cells (Bregs). The cells were detected by a Beckman coulter Navios flow cytometer and analyzed by Kaluza analysis software version 2.1.

### 2.5 Safety follow-up

The side effects of Peg-G-CSF and G-CSF mobilization were evaluated with a questionnaire until 2 weeks after apheresis. Pain scores were assessed on the count of numerical rating scale (NRS): painless (0), mild pain (1 ~ 3), moderate pain (4 ~ 6), severe pain (7 ~ 10). Safety was evaluated based on changes from baseline in the medical history, blood test and biochemical index measurements, and physical examination findings. The donors were followed up for 1 year.

### 2.6 Outcome of recipients

GVHD diagnosis, staging and organ specificity for recipients were determined according to the modified Glucksberg criteria and 2014 National Institutes of Health (NIH) consensus criteria (15, 16). The day of neutrophil engraftment was defined as the first of 3 days with an absolute neutrophil count of 0.5×10^9^/L after stem cell infusion. The day of platelet engraftment was defined as the first 7 days with a platelet count of 20×10^9^/L without platelet transfusions. Disease relapse was defined via cell morphology, cytogenetics or molecular biology or by evidence of leukemic cells in the BM or other extramedullary organs (17). PFS was defined as the shortest interval between HSCT and relapse or non-relapse mortality or the last follow-up. OS was defined as the interval between HSCT and death. GRFS was defined as being alive without grade III-IV aGVHD, severe extensive cGVHD requiring systemic immune-suppression treatment nor disease relapse at any time point.

### 2.7 Statistical analysis

SPSS (version 20.0, IBM) was used to perform all statistical analyses. Chi-squared tests were used for the baseline and GVHD incidence characteristics between the Peg-G-CSF group and the G-CSF group, and the Mann-Whitney U test was used for the continuous variables due to the skew distribution. OS and PFS were estimated using Kapan-Meier analysis. Log-rank tests were used to compare the OS and PFS curves between the two groups. Logistic regression analysis was used to analyze related factors. The proportional hazards method was used to estimate the cumulative incidence of relapse and NRM, GVHD. Relapse and NRM were competing risks for each other.

## 3 Results

### 3.1 Donor characteristics

The detailed characteristics of the 192 donors in the two groups were not significantly different (P>0.05, **Table 1**). The majority of donors in both groups were male. The median weight was approximately 60 kg, which was higher than that of the recipients in both groups. COBE Spectra was used for HSCs collection in most donors, and thirty-four donors used COE.TEC for HSCs collection. There was no difference in machine for HSCs collection between two groups (P = 0.450, **Table 1**). The processed blood volume and apheresis time in each group were no differences (P>0.1, **Table 1**).

**Table 1:** Characteristics of donors in two groups.

Parameter	Peg-G-CSF(n=96)	Parameter	G-CSF(n=96)	P
Median age, y (range)	38 (18-55)	Median age, y (range)	37 (19-55)	0.903
Male/female, n	67/29	Male/female, n	63/33	0.537
Median weight (kg, range)	65 (50-91.2)	Median weight (kg, range)	62 (50-88.6)	0.180
HLA match, n		HLA match, n		0.878
matched	32	matched	31	
Haplo-identical	64	Haplo-identical	65	
Donor-recipient gender match, n		Donor-recipient gender match, n		0.340
Male-male	32	Male-male	39	
Male-female	35	Male-female	24	
Female-female	11	Female-female	15	
Female-male	18	Female-male	18	
ABO match, n		ABO match, n		0.246
Matched	49	Matched	57	
mismatched	47	mismatched	39	
Leukapheresis device				
COBE spectra	81	COBE spectra	77	0.450
Median blood volumes (L)	13.6	Median blood volumes (L)	13.2	0.175
Average apheresis time (minutes)	313	Average apheresis time (minutes)	316	0.766
Two days collections, n	16	Two days collections, n	20	0.579
Median CD34 number on day5 (/uL, rang)	60.1 (14.8-219.4)	Median CD34 number on day5 (/uL, rang)	60.2 (7.8-275.8)	0.438
Median total nucleated cells/kg (×10^8^,rang)	13.3 (6.8-44.3)	Median total nucleated cells/kg (×10^8^,rang)	13.2 (6.4-36.8)	0.174

### 3.2 Peripheral blood cell counting analysis

**Figure 2:**
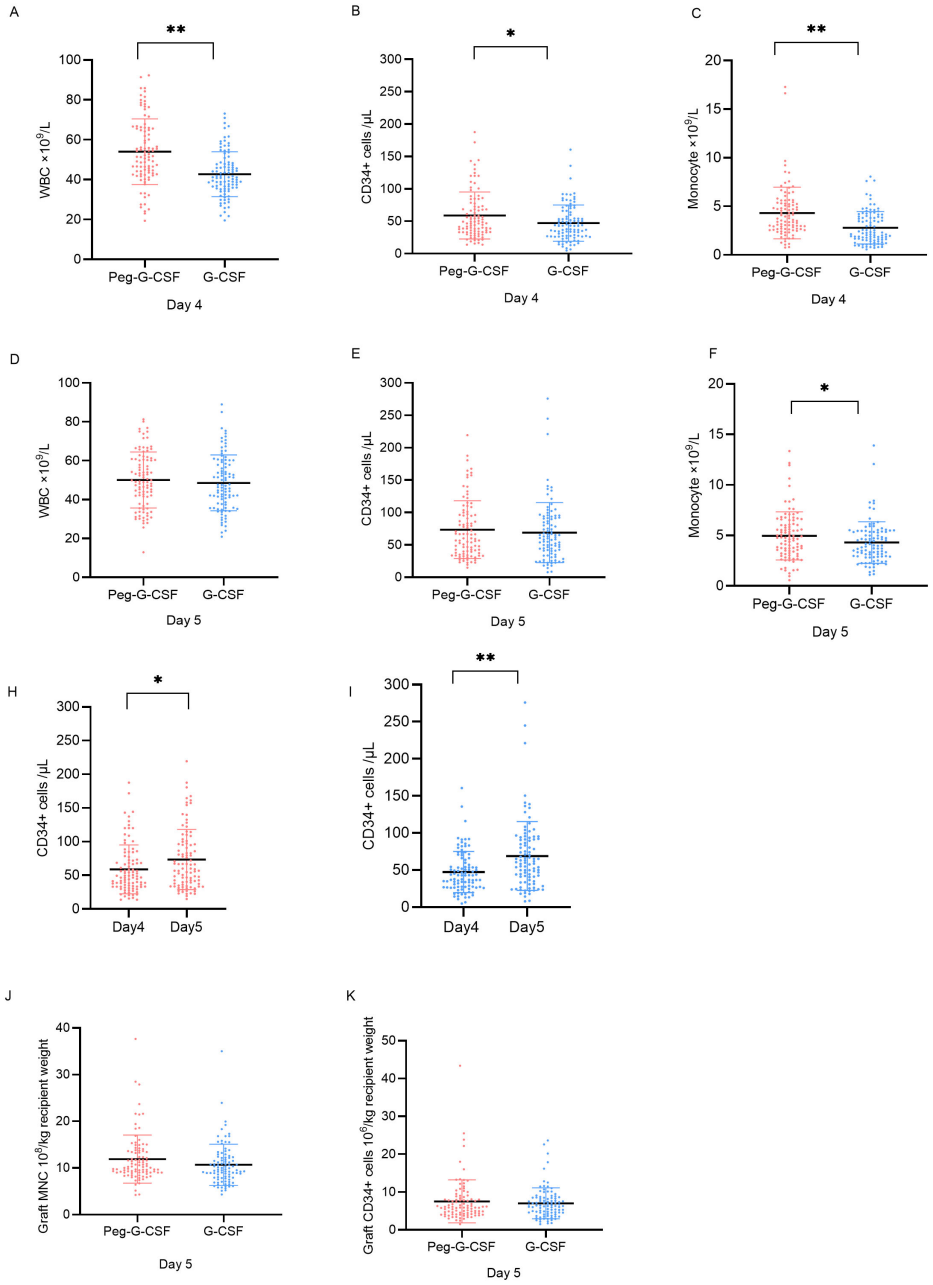
Efficiency analysis of hematopoietic stem cell mobilization after Peg-G-CSF and G-CSF administration between two groups. Peripheral blood analysis on day 4: **(A)** WBC, **(B)** CD34+ count and **(C)** monocyte count; Peripheral blood analysis on day 5: **(D)** WBC, **(E)** CD34+ cell count and **(F)** monocyte count; **(H)**.CD34+ cell count in the Peg-G-CSF group; **(I)** CD34+ cell count in the G-CSF group; **(J)** MNC and **(K)** CD34+ cell yield. Red spots represent mobilization with Peg-G-CSF, blue spots represent mobilization with G-CSF. ^*^P<0.05,^**^P<0.01;WBC: white blood cell;MNC: mononuclear cell.

Compared with the G-CSF mobilization, the upward trend in the WBC, CD34^+^ cell and monocyte counts in the peripheral blood were observed after mobilization with Peg-G-CSF (**Figure 2**). The median CD34^+^ cell count in the peripheral blood of donors were 47.7/µL and 42.9/µL in the Peg-G-CSF group and G-CSF group on day 4, respectively (P = 0.027). Nevertheless, the median CD34^+^ cell count on day 5 in the Peg-G-CSF group was similar to that in the G-CSF group (60.1/µL vs. 60.2/µL, P = 0.438). In addition, the peak value of circulating CD34^+^ cell count occurred on day 5 in both groups.

Similarly to CD34^+^ cell count, the median WBC of donors was significantly higher in the Peg-G-CSF group than in the G-CSF group on day 4 (51.6×10^9^/L vs. 41.2×10^9^/L, P<0.01). However, the advantage of WBC in the Peg-G-CSF group disappeared on day 5 (median: 49.2×10^9^/L vs. 46.8×10^9^/L, P = 0.397). The median monocyte count in the peripheral blood of the Peg-G-CSF group was observed to be much higher than those in the G-CSF group during the period of mobilization(P = 0.021).

Flow cytometry analysis of the graft was performed on day 5 to measure the lymphocyte subsets in the two groups. The median absolute number of CD3-CD19+ B cells in the Peg-G-CSF group was lower than that in the G-CSF group (15.9×10^9^/L vs. 18.9×10^9^/L, P = 0.047), but the absolute number of T lymphocyte did not differ significantly. There were no differences in the graft after mobilization in either the Peg-G-CSF group or the G-CSF group with respect to the median absolute number of NK cells (10.9×10^9^/L vs.12.2×10^9^/L, P = 0.389), Tregs (3.9×10^8^/L vs. 4.2×10^8^/L, P = 0.624) and Bregs (0.1×10^8^/L vs. 0.2×10^8^/L, P = 0.373).

### 3.3 Mobilization efficacy and graft products

As the CD34^+^ cell count peaked in both groups, PBSCs were collected on day 5. The percentage of donors who obtained the optimal number of CD34^+^ cells ≥4×10^6^/kg recipient weight after a single apheresis procedure was 83.3% in the Peg-G-CSF group and 79.2% in the G-CSF group, and there was no difference in optimal mobilization between the two groups (P = 0.460). Almost all donors from the two groups were able to reach the minimal collection number of CD34^+^ cells≥2×10^6^/kg recipient weight (99.0% in the Peg-G-CSF group vs. 96.9% in the G-CSF group, P = 0.178) with a single apheresis procedure. The mobilization efficiency of Peg-G-CSF was comparable to that of G-CSF. Only 5 donors in the Peg-G-CSF group received additional G-CSF, because their circulating CD34^+^ cell count were ≤20/µL on day 4. The number of CD34^+^ cells reached ≥ 4×10^6^/kg recipient weight in three of the five donors in a single apheresis procedure. In the peg-G-CSF group and the G-CSF group, the CD34^+^ cell output of 16 and 20 donors did not reach the primary endpoint. The median of CD34^+^ cells/µL in peripheral blood was 37.0/µL (19.4-66.9) and 23.7/µL (7.8-128.2), respectively. The pre-procedure CD34^+^ cell count in peripheral blood was a significant difference between the good and poor mobilizers (P = 0.000).

Among the grafts, the median yield of CD34^+^ cell based on recipient weight collected by a single apheresis procedure in the Peg-G-CSF group was comparable to that in the G-CSF group at 5.9×10^6^/kg (range: 1.5-43.3×10^6^/kg) vs. 6.3×10^6^/kg (range: 1.5-23.6×10^6^/kg) (P = 0.987, **Figure 2**). The median yield of mononuclear cell in the Peg-G-CSF grafts was 10.3×10^8^/kg recipient weight (range: 4.2-37.6×10^8^/kg), which was higher than that in the G-CSF group (9.7×10^8^/kg; range: 4.3-35.0×10^8^/kg) (P = 0.050, **Figure 2**).

### 3.4 Safety of mobilization

A questionnaire was designed for donors to measure side effects. The total adverse event between the two groups was not statistically different (P = 0.624, **Table 2**). Similar to the side effects of G-CSF, bone pain and headache are the two main side effects of Peg-G-CSF. The incidence of bone pain and headache in the Peg-G-CSF group were 67.7% and 42.7%, respectively. As recorded on the NRS, a few donors who were injected with Peg-G-CSF and G-CSF experienced mild pain. Pain scores and transient bone pain were similar in the two groups. None of the donors were treated with pain medication. Other complaints observed in this study included sleeplessness, tiredness and loss of appetite, and the relatively low incidence of these side effects did not differ between the groups. In addition, no cases of splenomegaly were found in the physical examination. Biochemical index measurements were changed during mobilization. The mean level of lactate dehydrogenase(LDH) after mobilization increased 3-fold in the Peg-G-CSF group compared with baseline levels and increased 2-fold in the G-CSF group (P<0.01).The mean level of alkaline phosphatase (ALP) after mobilization increased by approximately 2-fold in both groups (P<0.01). The higher ALP likely reflects the higher WBC in the peg-G-CSF and the G-CSF group on day 4. There was a linear correlation between ALP and WBC (P = 0.007). The elevation of ALP and LDH did not require medical treatment, and these levels gradually returned to their normal values after cell collection. No serious or fatal adverse events occurred in donors of either group, and no donors died due to adverse reactions.

**Table 2:** Side effects in two groups.

Parameter		Peg-G-CSF	G-CSF	P value
Symptom	intensity	N (%)	N (%)	
Total AE	/	69(71.8)	72(74.2)	0.624
Headache	total	41(42.7)	34 (34.4)	0.824
	mild	34 (35.4)	28 (29.2)	
	moderate	7 (7.5)	5(5.2)	
Bone pain	total	65 (67.7)	57 (59.4)	0.230
	mild	60 (62.5)	51 (53.1)	
	moderate	5 (5.2)	6 (6.3)	
Other complaints	Sleeplessness	3 (3.1)	4 (4.2)	1.000
	Tiredness	5 (5.2)	2 (2.1)	0.441
	Loss of appetite	6 (6.3)	4 (4.2)	0.516
Mean LDH level (IU/L)	609 (368.2-1039)		440.65 (332.7-578.1)	0.164
Mean ALP level (IU/L)	201.8 (137.4-504.3)		162.6 (98.2-299.4)	0.016

AE, adverse event; LDH, lactate dehydrogenase; ALP, alkaline phosphatase.

There was no significant difference in the timepoint at which the WBC returned to a normal value (P = 0.932). The WBC of 82 (85.4%) donors in the Peg-G-CSF group recovered within 1 week after PBSC leukapheresis, 12 donors recovered within 2 weeks, and 2 donors did not undergo a routine blood examination. In the G-CSF group, the hemogram values returned to baseline values within 1 week after collection in 79 (82.3%) donors and within 2 weeks in 12 donors; 5 donors were not assessed. The results showed that the recovery time with Peg-G-CSF mobilization did not extend further than that with G-CSF mobilization.

### 3.5 Recipient characteristics and engraftment

All enrolled recipients were diagnosed with acute leukemia. The gender of the recipients in the Peg-G-CSF group and the G-CSF group was not different(P>0.05). The median weight of recipients in the two groups was 55.4 kg (range 11.5–91 kg) and 53.6 kg (range 10–81 kg), respectively (P = 0.422). The median weight ratio between the donors and the recipients in the two groups was 1.16 and 1.15 (p = 0.999). The percentage of recipients in the two groups that received haplo-HSCT were 66.7% and 67.7%, respectively (P = 0.878). The median age of the recipients was 26 years and 29 years in the Peg-G-CSF group and the G-CSF group, respectively (P = 0.813).

In the G-CSF group, 1 recipient failed to achieve hematopoietic reconstitution, and 1 patient failed to achieve platelet reconstitution. However, in the Peg-G-CSF administration group, only 1 recipient did not reach hematopoietic reconstitution. The time to neutrophil engraftment was 15 days in both groups. The median time for PLT engraftment was 16 days and 15 days after mobilization with Peg-G-CSF and G-CSF, respectively.

### 3.6 GVHD

**Figure 3:**
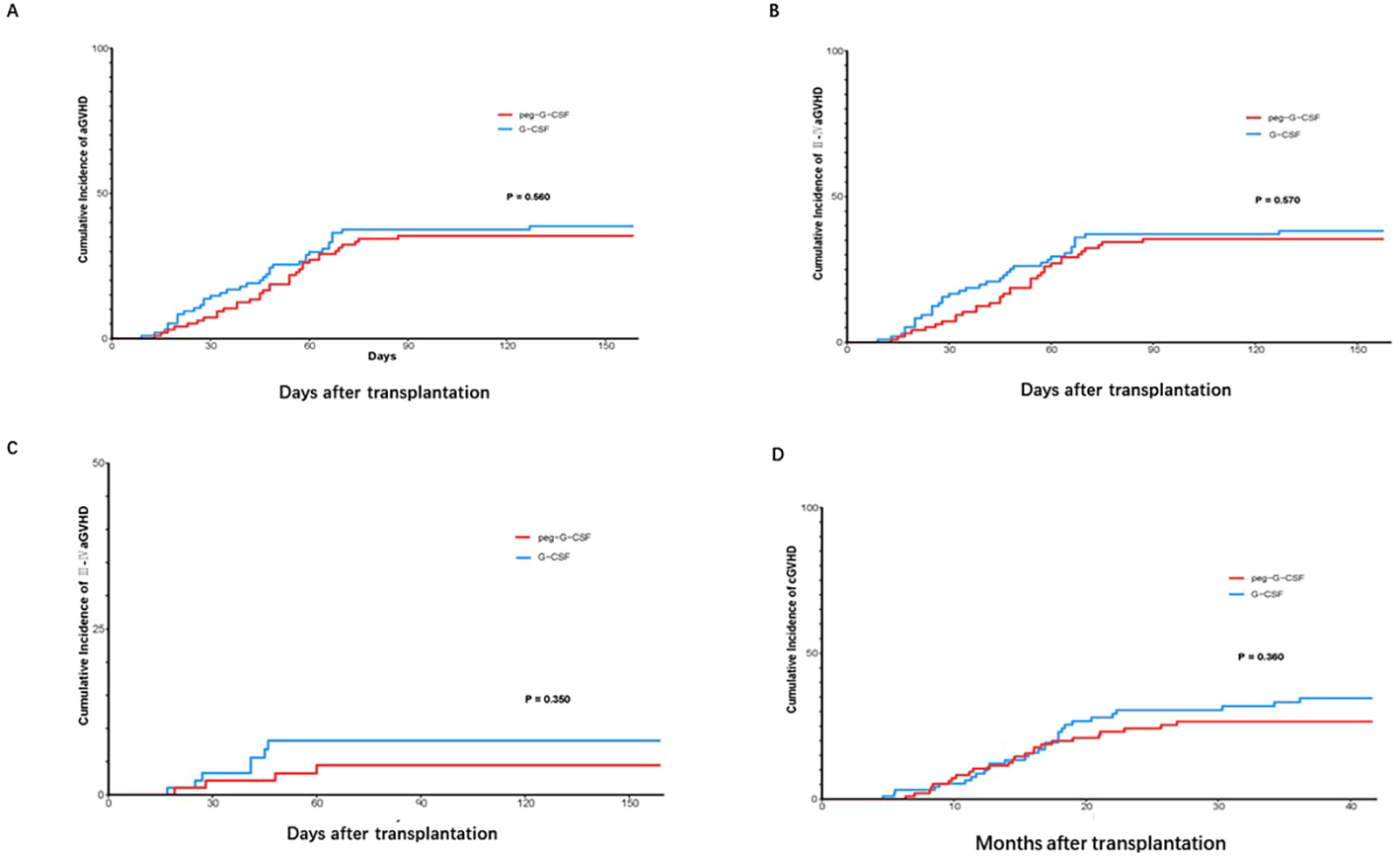
Cumulative incidence of GVHD. **(A)** Cumulative incidence of aGVHD. **(B)** Cumulative incidence of II-IV aGVHD. **(C)** Cumulative incidence of III-IV aGVHD. **(D)** Cumulative incidence of cGVHD. GVHD: graft-versus-host disease; aGVHD, acute graft-versus-host disease; cGVHD, chronic graft-versus-host disease.

No significant differences in the rates of aGVHD and cGVHD between recipients in two groups were noted. The 100-day cumulative incidences of aGVHD among the recipients in the Peg-G-CSF group (36.5%) was similar to that among those in the G-CSF group (39.7%, P = 0.560, **Figure 3A**). The grades II to IV aGVHD was 36.5% vs. 39.3% (**Figure 3B**), whereas that of grades III to IV aGVHD was 5.87% vs 9.87% (**Figure 3C**). In the Peg-G-CSF group, the cumulative incidences of cGVHD was 27.8%, while the cumulative incidences of cGVHD occurred in 36% of the recipients in the G-CSF group (P = 0.360 **Figure 3D**). The common sites of cGVHD were skin mucocutaneous and liver.

### 3.7 Recipient outcomes

**Figure 4:**
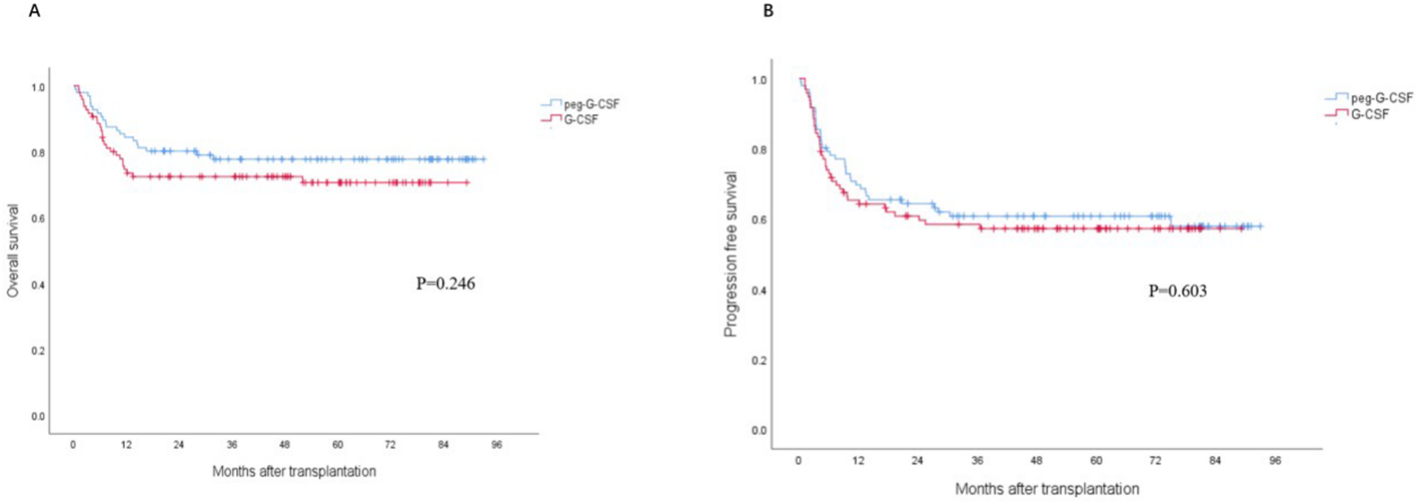
Kaplan-Meier estimated overall survival and progression-free survival of patients in the two groups. Overall survival **(A)**, Progression-free survival **(B)**.

Recipients’ survival was evaluated at a median follow-up timepoint of 47 months (range of 1.1–93 months). The estimated 5-year OS was 77.6%, which was quite similar to that in the G-CSF group (70.6%, p=0.246, **Figure 4** A). The 5-year PFS were 57.9% and 57.2% in the Peg-G-CSF group and the G-CSF group, respectively (**Figure 4** B). After allo-HSCT, 16 recipients died because of recurrence in the Peg-G-CSF group, whereas 14 relapse recipients died in the G-CSF group. The cumulative incidence of 3-years relapse was 17.5% vs 15.5%. The cumulative NRM of 3-years were 6.76% and 15.2% (P = 0.099), respectively. In addition, the 3-years cumulative GRFS rates were 51.52% and 45.92% (P = 0.621) in the Peg-G-CSF group and the G-CSF group.

### 3.8 Multivariable outcome analysis

To estimate the factors with regard to efficiency mobilization, logistic regression was used to identify potential clinical predictors for CD34^+^ cell yield. For univariate analysis, we examined the relationship between CD34^+^ cell yield and the following variables: mobilization mode, donor age (<38 vs. ≥38 years), donor weight, WBC, monocyte count and CD34^+^ cell count before apheresis. Univariate analysis revealed that WBC on day 4-5,monocyte count on day 5,CD34^+^ cell count on day 4–5 were significant factors affecting CD34^+^ cell yield(P<0.05). However, only CD34^+^ cell count on day 5 had an influence on CD 34^+^ cell yield in multivariate analysis (P = 0.002).

## 4 Discussion

Peg-G-CSF is a long-acting agent that is widely used to prevent febrile neutropenia after chemotherapy in patients (18). In recent years, preliminary data from animal studies and normal human volunteers also suggest that Peg-G-CSF may be able to mobilize peripheral blood progenitor cells with a single subcutaneous injection without added toxicity, thus avoiding the need for repeated injections (13, 19). However, there is still insufficient evidence of feasibility for Peg-G-CSF mobilization, especially in terms of allo-HSCT. Consequently, we designed a multicenter RCT to verify the role of Peg-G-CSF in PBSC mobilization in allo-HSCT healthy donors. Previous studies showed that donors could be treated with single doses of 6–15 mg Peg-G-CSF-induced mobilization, and most of them chose 12 mg Peg-G-CSF because of the efficiency and steady-state mobilization (13, 20, 21). Therefore, we chose a single fixed dose of 12 mg Peg-G-CSF and investigated the effects of mobilization in healthy donors.

In addition to the dose of Peg-G-CSF, there was no agreement on the timing of the collection. Molineux et al. reported that the maximum concentration of peripheral CD34^+^ cells after 300 µg/kg Peg-G-CSF-stimulated administration was detected on day 4 in healthy volunteers (19). However, another study demonstrated that healthy volunteers with 12 mg Peg-G-CSF showed a sufficient increase in CD34^+^ cells, with a peak value of circulating CD34^+^ cells observed on day 5 (13). A single-arm phase 2 study evaluated the efficacy and safety of pegfilgrastim for PBSC mobilization in healthy volunteers. In the evaluation phase, successful CD34^+^ mobilization was achieved in all 23 subjects. The mean peripheral blood CD34^+^ cells count peaked on day 5 (22). The different results from previous reports may have been due to the small sample size and different drug doses. The kinetics of circulating CD34^+^ cells were also evaluated in our study to guide the scheduling of leukapheresis procedures. We observed that peak concentrations of circulating CD34^+^ cells in the Peg-G-CSF and G-CSF groups appeared on day 5, which were consistent with our retrospective study (11). We further demonstrated the dynamics of drug administration throughout an RCT. The apheresis procedure was performed on day 5 after initiation of Peg-G-CSF and G-CSF administration because at that time, the concentration of stem cells in the peripheral blood peaked.

The optimal number of CD34^+^ cells after one apheresis procedure is recommended to be 4 to 5×10^6^ cells/kg recipient weight by ABMT (12). Hill reported a phase I/II study in which 92.3% (12/13) of donors with 12 mg Peg-G-CSF-induced mobilization achieved CD34^+^ cell ≥4×10^6^/kg recipient weight after a single apheresis procedure, while the percentage of 19 donors using G-CSF was 68.0%, which indicated that Peg-G-CSF may be superior to traditional G-CSF in mobilization efficacy (23). In this RCT, we found that 83.3% of donors in the Peg-G-CSF group and 79.2% of donors in the G-CSF group yielded the optimal number of CD34^+^ cells (≥4×10^6^/kg recipient weight) in a single apheresis procedure. Peg-G-CSF mobilization and classic G-CSF mobilization were therefore comparable. Our study results were inconsistent with Hill’s, which may be due to the small sample size and non-RCT in their study, thus causing statistical deviation.

In terms of safety, the side effects of Peg-G-CSF mobilization were not different from those of G-CSF. The major complications of Peg-G-CSF are bone pain and headache. In our study, all the side effects were transient and usually mild in severity. Elevations in liver enzymes have been reported in some trials (11, 23–25). Peg-G-CSF can cause a mean 4-fold increase in both ALP and LDH activity, and increases were observed in all donors. Our results also showed that most donors exhibited increased LDH and ALP levels after Peg-G-CSF and G-CSF administration. Cell proliferation is known to result in elevated LDH (26); however, transient increases in LDH and ALP do not damage the body or necessitate medical treatment. These adverse reactions are consistent with a recent study report in Japan (22).Excessive increases in WBC might be associated with splenic enlargement and the potential risk of splenic rupture. A study reported that rupture of the spleen occurred in a donor whose WBC was >50×10^9^/L after the injection of G-CSF (27). Some medical experts are concerned that mobilization with Peg-G-CSF could cause excessive increases in WBC for a long period and raise the risk of splenomegaly. Although there was no association between the increase in WBC and the recovery time in our study, and although splenic rupture caused by Peg-G-CSF mobilization has not been reported in allo-HSCT donors to date, we must still pay special attention to the possible occurrence of splenomegaly. Splenic enlargement did not occur during mobilization, as assessed by physical examination in our trial. This might be explained by Peg-G-CSF being eliminated by cellular uptake and intracellular degradation through the G-CSF receptor with an increase in WBC (28). Therefore, compared with traditional G-CSF, a single dose of 12 mg Peg-G-CSF for mobilization is safe and has an acceptable and comparable toxicity.

Acute graft-versus-host disease remains one of the most frequent complications following allo-HSCT with high mortality (29). Both GVHD and GVL are immune reactions that are closely related to T cells. GVL and GVHD often occur together but not in complete harmony (30). Donor treatment with Peg-G-CSF may promote immune tolerance and decrease GVHD (31). Peg-G-CSF is markedly superior to standard G-CSF for preventing GVHD in animal models following allogeneic stem cell transplantation due to the generation of IL-10-producing Tregs. Modification of G-CSF by pegylation of the native cytokine results in the expansion and activation of donor invariant NK T cells, which significantly increase CD8+ T cell-mediated cytotoxicity and GVL effects after transplantation (32). However, inconsistent with the conclusions of previous studies, our results showed that the incidences of aGVHD and cGVHD were similar between the two groups. Moreover, the GVHD staging and target organ specificity were quite similar within the two groups, the proportion of moderate cGVHD incidence in the Peg-G-CSF group seemed to be downtrend. Lymphocyte subsets play an important role in GVHD onset and development. Tregs function to suppress alloreactive immune responses and are key mediators of immune tolerance (33). A subset of Bregs negatively regulate T cell immune responses in mice through the secretion of regulatory cytokines such as IL-10 and direct cell-cell contact (34). Invariant natural killer T cells can ameliorate GVHD through the activation of Tregs after their own activation with α-galactosylceramide (α-GalCer) or adoptive transfer (35). Our results indicated that the T lymphocyte of graft was not significantly different between the two groups. The similar incidence of GVHD was due to the fact that there was no difference in the NK cell, Tregs, Bregs between the Peg-G-CSF and G-CSF group. The subgroup variation was consistent with the results of Li’s report (36).

Age was reported to be an important factor in stem cell collection. A significantly higher number of CD34^+^ cells with the target was achieved more frequently in young donors (≤38 years old) (37). Unlike reports from other authors (37–39), our study showed that there was no correlation between age, WBC and monocyte count and CD34^+^ mobilization efficacy in the univariate analysis. A peripheral CD34^+^ cell count > 20/μL maximizes the likelihood of adequate cell collection with fewer apheresis session. In multivariate analysis the CD 34^+^ count on the fifth day was still the only important independent factor for predicting the production of single mining, which was consistent with a previous study (40).

Our study did have certain limitations. First, younger male patients are associated with a higher yield of CD34^+^ cells. In the case of similar HLA compatibility, we preferred male donors in our study. Second, there is an absence of data about long term safety of PEG in health donors. Further careful evaluation of safety issues is necessary. Third, We have not yet fully delineated the role of specific immune regulators (e.g., MDSCs), limiting our ability to accurately link immune modulation to clinical outcomes in recipients.

## 5 Conclusion

Our study is the first multicenter randomized control trial comparing mobilization by Peg-G-CSF and that by G-CSF in an allogenic setting. We showed that, compared with traditional G-CSF, a single injection of Peg-G-CSF had equal efficacy and safety for mobilization and circumvented the burden of daily injections and apheresis procedures in donors. Peg-G-CSF induced mobilization may be an alternative approach to G-CSF-induced mobilization, which needs to be further evaluated and expanded.

## Data Availability

The raw data supporting the conclusions of this article will be made available by the authors, without undue reservation.
